# Evaluation of quadriceps muscle cross-sectional area using an ultrasonic diagnostic equipment with a wide field of view

**DOI:** 10.1371/journal.pone.0311043

**Published:** 2024-09-24

**Authors:** Yasumoto Matsui, Marie Takemura, Yasuo Suzuki, Tsuyoshi Watanabe, Keisuke Maeda, Shosuke Satake, Hidenori Arai

**Affiliations:** 1 Center for Frailty and Locomotive Syndrome, Hospital, National Center for Geriatrics and Gerontology, Obu, Japan; 2 Department of Human Care Engineering, Nihon Fukushi University, Mihama-cho, Japan; 3 Department of Orthopedic Surgery, Hospital, National Center for Geriatrics and Gerontology, Obu, Japan; 4 Nutrition Therapy Support Center, Aichi Medical University Hospital, Nagakute, Japan; 5 Department of Geriatric Medicine, Hospital, National Center for Geriatrics and Gerontology, Obu, Japan; 6 National Center for Geriatrics and Gerontology, Obu, Japan; Ritsumeikan University, JAPAN

## Abstract

Skeletal muscle index measurement via dual-energy X-ray absorptiometry or bioelectrical impedance analysis is used to evaluate muscle mass when diagnosing sarcopenia. However, inherent challenges exist with these methods. We previously focused on muscle mass evaluation in the quadriceps femoris by using computed tomography (CT). In this observational study, we utilized a new ultrasound device with a wide field of view that can obtain CT-like images and investigated its usefulness. Reproducibility was investigated by calculating the intra- and inter-examiner intraclass correlation coefficient (ICC) by using three examiners and performing five measurements in 12 participants. In 123 participants (48 men, 75 women, mean age 78.2 ± 8.1 years), we investigated the correlation between the quadriceps cross-sectional area measured with supine ultrasound and CT images as well as between supine and sitting ultrasound images. Unadjusted, age-adjusted, and age–sex-adjusted correlations were calculated. Reproducibility was excellent (intra-examiner ICC[1,1]: 0.978, 0.987, and 0.994; inter-examiner ICC[2,1]: 0.993). The unadjusted, age-adjusted, and age–sex-adjusted correlations between the quadriceps cross-sectional area measured using supine ultrasound and CT were 0.949, 0.940, and 0.894, respectively. For sitting ultrasound, the corresponding values were 0.958, 0.953, and 0.912, respectively. Correlations between the supine and sitting ultrasound measurements were also good, with corresponding values of 0.952, 0.945, and 0.904, respectively. The tested ultrasound device showed excellent measurement reproducibility and had good correlations with CT images. Further studies with an increased numbers of clinical cases and additional evaluations should allow the device to become a screening tool for diagnosing sarcopenia.

## Introduction

In today’s aging society, the extension of healthy life expectancy is an important issue. To address this, it is necessary to assess sarcopenia appropriately and at an early stage. Sarcopenia has been reported to increase the risk of decreased physical function, hospitalization, and death [1]. Sarcopenia was originally defined by Rosenberg in 1989 from the Greek words "sarx" (muscle) and "penia" (loss) as the age-related decrease in skeletal muscle mass [2]. The diagnosis of sarcopenia was made in 1998 by Baumgartner et al. [3], who defined sarcopenia as mean skeletal muscle mass index (SMI) of two standard deviations or less when measured using dual-energy X-ray absorptiometry (DXA) in young people. Since then, SMI has been used to determine sarcopenia. However, previous studies have reported that muscle strength is more closely related to falls and motor function than muscle mass [4,5]. Therefore, muscle strength and physical ability should also be included when diagnosing sarcopenia. The European Working Group on Sarcopenia in Older People defined sarcopenia as "low muscle mass and low muscle function (strength or performance)" [6], thus facilitating uniformity in diagnostic standards. The diagnostic algorithm was revised in 2018 [7]. Additionally, in 2014, the Asia Working Group for Sarcopenia established diagnostic criteria for sarcopenia on the basis of data obtained from Asian countries [8]. In 2019, diagnostic algorithms, protocols, and some reference values were revised with the introduction of the term "possible sarcopenia," which is defined as a decline in physical muscle mass, but it is known that this index does not decrease with age in Asian women [9,10]. Bioelectrical impedance analysis (BIA), which is another method for assessing muscle mass, has some limitations, namely, it is device and population specific and is highly influenced by the body’s hydration status, thus resulting in the underestimation of muscle mass [11].

As described above, the current approach to diagnose sarcopenia involves measuring the muscle mass in the entire limb [7,12]. However, given the region-wise variability in muscle loss associated with aging, measuring muscle mass in regions that are assumed to be more strongly related to physical function may be more useful. Considering that the amount of muscle loss associated with aging in the lower limbs is twice that of the upper limbs [13,14], it is thought that the lower limbs are more susceptible to the effects of aging [15]. Within the lower limb, the thigh is a region where age-related muscle loss is significant and is often used in related research [16]. We have been focusing on quadriceps as it is known to decrease at similar rate to trunk muscles in a human body [17]. The quadriceps femoris is an anti-gravity muscle that is important for daily activities, such as standing up and climbing/descending stairs. Thus, we have been evaluating the quadriceps femoris muscle and conducted quantitative evaluations by using computed tomography (CT) images, which are accurate and can be measured quickly [18,19]. In the general population, the CT images of the midthigh region show that the quadriceps muscle decreases not only quantitatively but also qualitatively (increase in intramuscular fat) because of aging [20]. Muscle quality is also associated with knee extension strength independently of muscle mass [20]. In addition, we found that in elderly people who tend to be frail, muscle strength is most closely related to muscle cross-sectional area, whereas physical function is more closely related to muscle quality [21] and we recently reported that the quadriceps muscle cross-sectional area is useful for diagnosing sarcopenia [22]. However, CT has problems such as high cost, space limitations, requirement of a measurement specialist, and exposure to radiation. Therefore, it would be desirable to develop a simple measurement device that can display a wide cross-section similar to CT. Ultrasonography is a simple method for evaluating muscle mass and quality. Using conventional ultrasound equipment, there have been several reports showing the utility of ultrasound for measuring the muscle thickness of specific components of the quadriceps femoris (including the rectus femoris, vastus intermedius [23–25], and vastus lateralis [26,27]) However, for clinical applications, a qualified examiner is necessary for measurements because of the requisite skill for ensuring reproducibility. Measurements are manually performed on the device screen; therefore, automation is lacking. Furthermore, the representation of muscle image is not easily comprehensible in terms of its shape. Therefore, it is still in the research stage and is rarely used in clinical settings.

The National Center for Geriatrics and Gerontology and Furuno Electric Co., Ltd. have been pursuing joint research to develop a new device that can easily and reproducibly measure the cross-sectional area of the quadriceps femoris.

In this study, we introduce a new ultrasonic measurement device that can visualize the quadriceps muscle over a wide area, similar to CT, and present the reproducibility of the measurement method. We aimed to assess the usefulness of this device, defined by the ability to measure the muscle cross-sectional area similarly to CT, as CT is considered the gold standard of muscle mass measurement by the European Working Group on Sarcopenia in Older People [6,8]. Additionally, we compared the cross-sectional area of the quadriceps in the supine position measured using ultrasound to that measured using CT of the same cross-section as well as to that using ultrasound in the sitting position, which would be easier to perform. These results demonstrate the usefulness of this device for evaluating muscle mass in elderly individuals who tend to be frail.

## Materials and methods

### Ultrasonic measurement equipment

We utilized the new ultrasonic muscle imaging system developed by Furuno Electric Co., Ltd. (Nishinomiya, Japan). The A correlation between the quadriceps cross-sectional area measured from CT and ultrasound images was evaluated at the National Center for Geriatrics and Gerontology.

**Fig 1:**
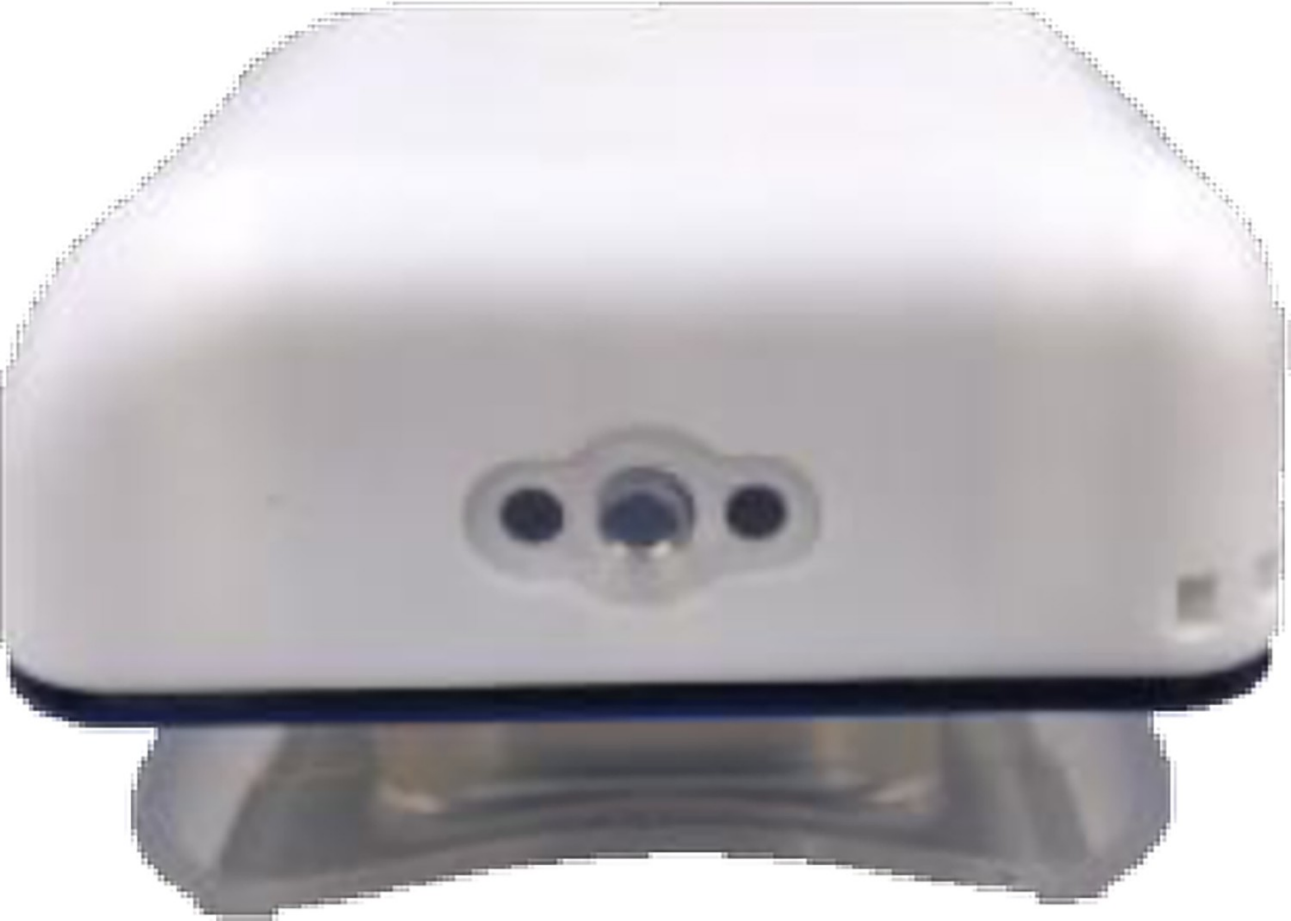
External view of the equipment. The device is designed to be compact and lightweight to facilitate measurements in various locations.

**Fig 2:**
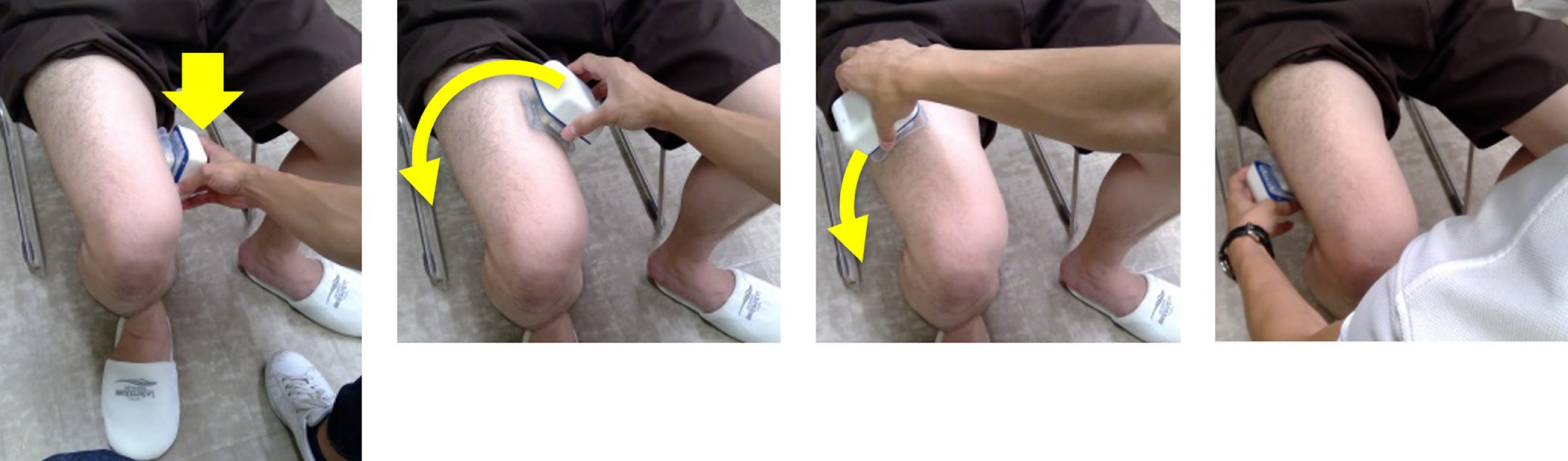
Measurement procedure. The device is positioned on the thigh. A complete measurement is obtained by sliding it over approximately two-thirds of the thigh’s circumference.

**Fig 3:**
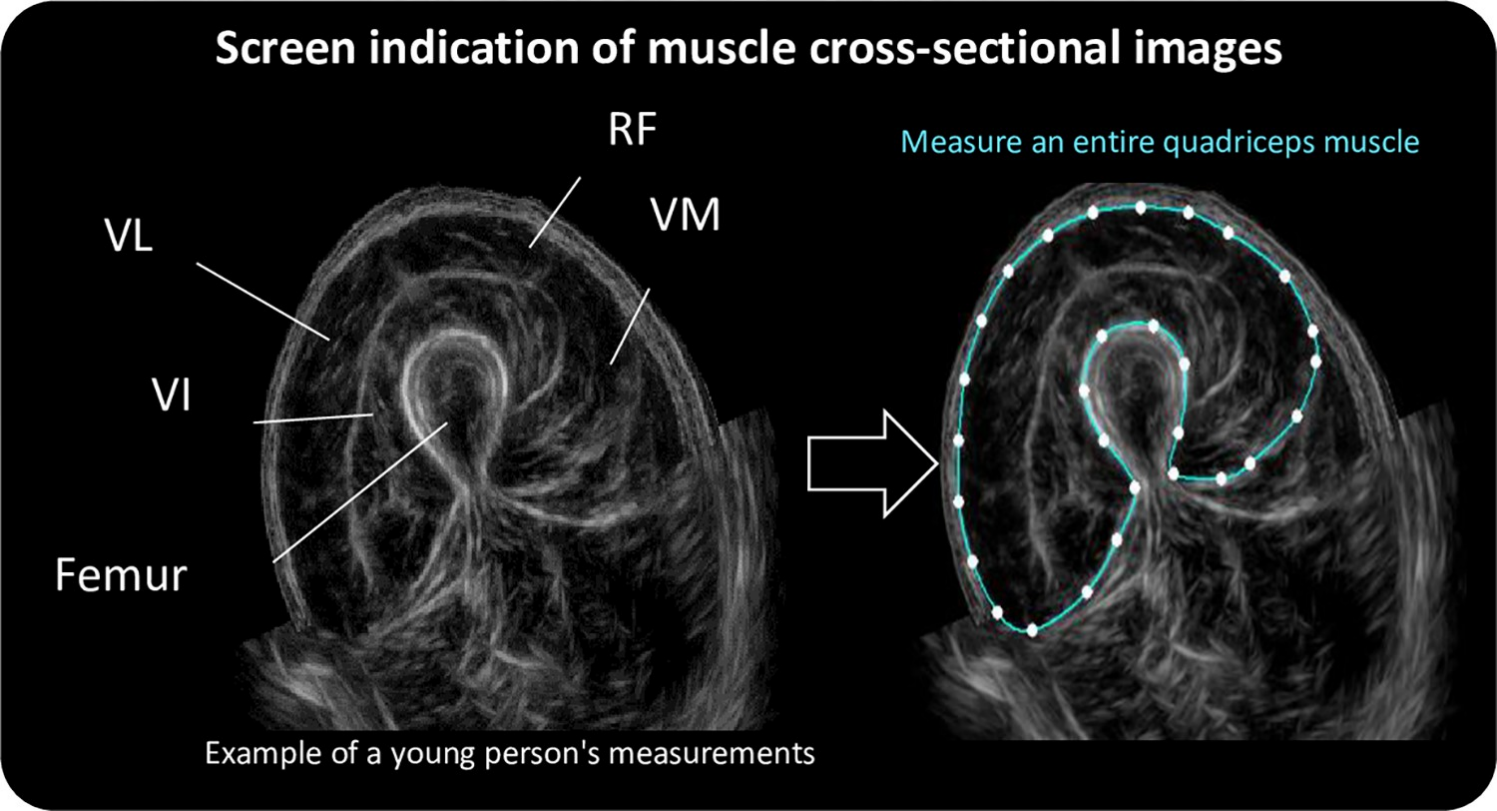
Cross-sectional muscle images. Illustration showing a young individual’s muscle measurement. RF: Rectus femoris; VM: Vastus medialis; VL: Vastus lateralis; VI: Vastus intermedius. Right: Entire quadriceps muscle encircled.

**Fig 4:**
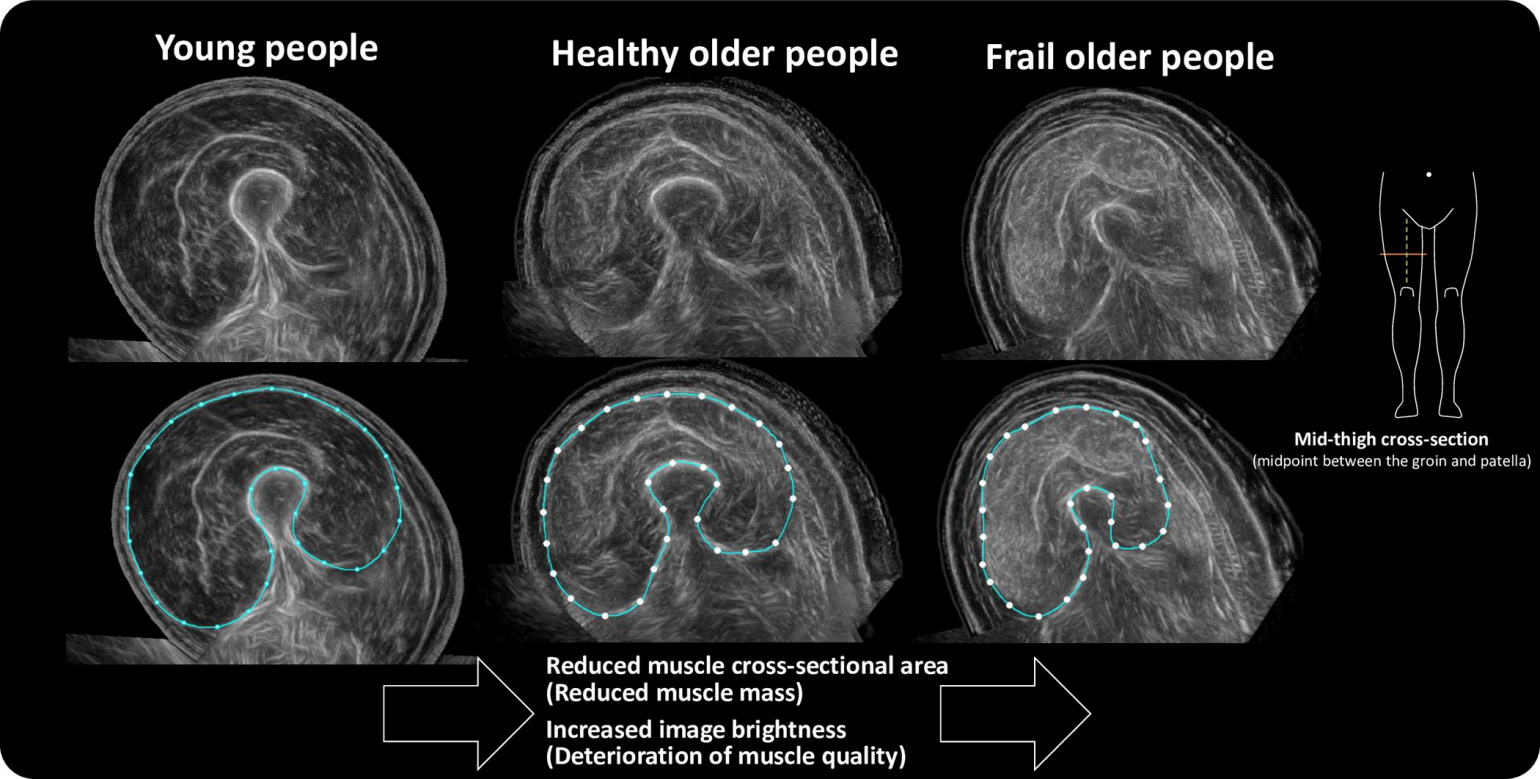
Characteristics of cross-sectional images of the quadriceps. Representative images of young adults (left), healthy older adults (middle), and frail older adults (right). A progressive decrease in muscle cross-sectional area and an increase in image brightness are observed, indicating deterioration of muscle quality in this sequence.

**Fig 5:**
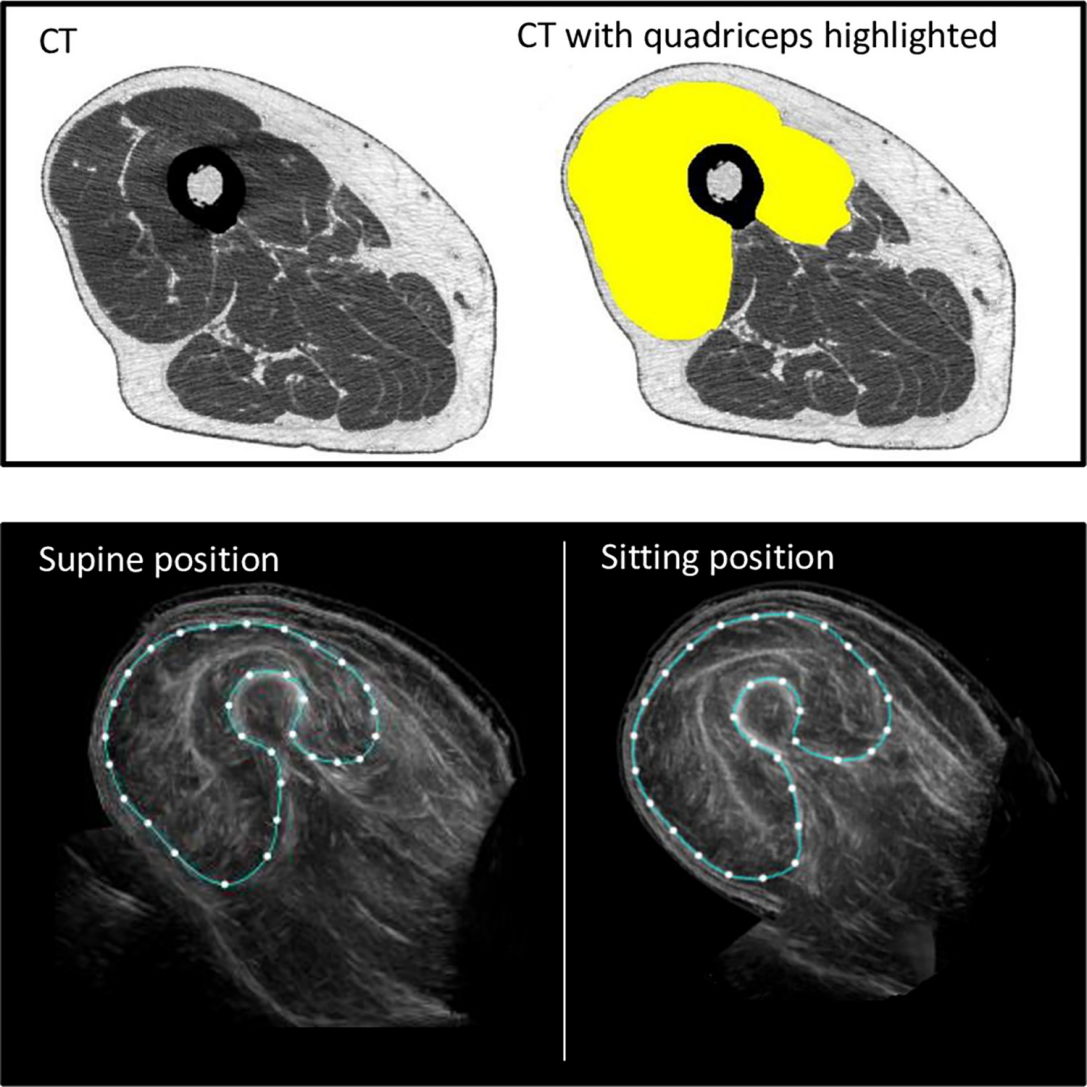
Three images of the same mid-thigh section using CT, supine ultrasound, and sitting ultrasound. Representative images of the same mid-thigh section using CT (upper), supine ultrasound (lower left), and sitting ultrasound young adults (lower right).

This ultrasonic muscle imaging system uses technology for panorama-synthesis-sector B-mode images; by applying it to the thigh and moving it in one direction along the skin, it is possible to display a muscle cross-sectional image similar to that of CT. Information such as the muscle cross-sectional area can be calculated by setting a region on the muscle cross-sectional image. Owing to the improvements, the device is small and lightweight (width 10 cm, weight approximately 260 g) and can be used anywhere (Fig 1). The probe was gently and slowly slid for approximately 10 s around two-thirds of the anterior thigh (Fig 2). Information was sent wirelessly to a Microsoft Surface Pro tablet placed nearby, and a cross-sectional muscle image (Fig 3) was automatically displayed within approximately 15 s. The quadriceps muscle boundaries were marked manually on the obtained image for cross-sectional area measurement. Typical images of healthy young, healthy old, and frail elderly individuals are shown in Fig 4. Representative images of the same mid-thigh section using CT, supine ultrasound, and sitting ultrasound young adults are shown in Fig 5.

### Measurement reproducibility

#### Verification of measurement reproducibility

Reproducibility was confirmed by comparing the five quadriceps cross-sectional area measurements in 12 participants (seven males and five females, average age 46.8 years, as shown in Table 1) using three examiners, and by determining the intraclass correlation coefficient (ICC). To eliminate the influence of manual positioning reproducibility, measurements were performed at the previously marked set positions. Measurements were performed without interval time to avoid the effects of posture positioning and diurnal and daily variations of the human body. This reproducibility was evaluated based on the technique used during measurement and the reproducibility of CSA analysis from the synthetic images obtained. As for the reproducibility of the synthetic image construction from ultrasound B-mode images, using the same image data ensures no operational errors, as the image is automatically and uniquely synthesized by the programmed image synthesis algorithm.

**Table 1:** Demographic information of the participants included in the reproducibility analysis.

	N	Age (years)		
		Min	Average	Max
All	12	27	46.8	60
Female	5	27	43.7	58
Male	7	33	49.8	60

#### Sample size and measurement details

**Fig 6:**
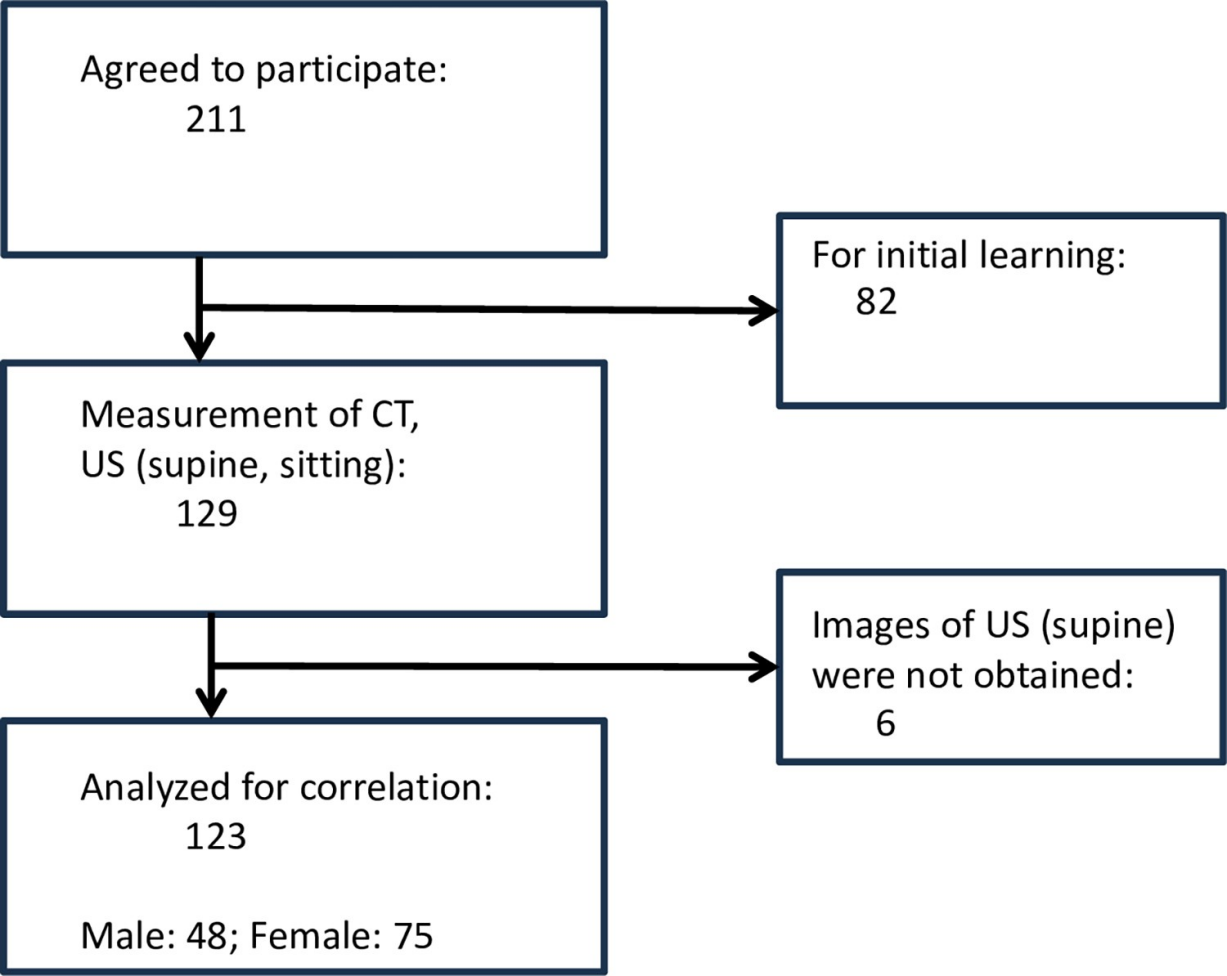
Participant recruitment flowchart.

Among the 211 frail locomotive syndrome registry (UMIN000048735) participants at the National Center for Geriatrics and Gerontology who consented to participate in the study, 82 subjects were excluded due to being used to check quadriceps muscle boundary evaluations made on ultrasound images by comparing and matching with the CT images for confirmation. Cross-sectional areas were calculated blindly from ultrasound images, and 123 subjects (48 men, 75 women, mean age 78.2 ± 8.1) were eligible for evaluation in both the sitting and supine positions. A flowchart of the recruitment process is shown in Fig 6.

The measurement site was a cross-section at the midpoint of a line connecting the upper pole of the patella to the center of the inguinal ligament. Ultrasound measurements were performed in two limb positions: lying supine on a bed and sitting on a chair. Both hip and knee joints were set at 90-degree flexion for the sitting position measurement posture. The CT device used was Aquilion CXL (CANON, Tochigi, Japan; settings: 135 kV, 150 mA; rotation time: 1 s; field of view: 4.0 mm) and SliceOmatic imaging software (version 5.0; Tomovision, Montreal, QC) was used to calculate the cross-sectional area of the entire quadriceps muscle from the CT images. The cross-sectional area was measured by outlining the four portions of the quadriceps muscle and the intermuscular fat. In order to minimize the effects of inter- and intra-day variations, CT and ultrasound were measured within one hour of the same day.

### Ethics

This study was conducted as a specific clinical study (N0057-4), registered under jRCT number jRCTs042210038, and approved by the Nagoya University Clinical Research Review Committee. This study was performed in accordance with the Declaration of Helsinki, and written informed consent was obtained from all participants.

### Statistical analysis

Measurement reproducibility was determined using intra- and inter-examiner ICC.

The ICC values were classified as poor (<0.5), moderate (0.5–0.75), good (0.76–0.9), and excellent (>0.9) as previously reported [28]. R version 4.2.2 (R Foundation for Statistical Computing, Vienna, Austria) was used for the reproducibility analysis. Correlations among ultrasound images measured in the supine and sitting positions and CT images were investigated using Pearson’s correlation coefficients (no adjustment) or partial correlation coefficients (with correction for age as well as for age and sex). SPSS version 24 (IBM Corp., Armonk, NY) was used for statistical analysis, and the significance criterion was p < 0.05.

## Results

Table 2 shows the reproducibility results.

**Table 2:** Reproducibility outcomes.

Intra-examiner	ICC(1,1)	
Examiner A	0.993	95% CI [0.985, 0.998]
Examiner B	0.996	95% CI [0.990, 0.999]
Examiner C	0.997	95% CI [0.992, 0.999]
Inter-examiner	ICC(2,1)	
	0.993	95% CI [0.981, 0.998]

ICC, Intraclass correlation coefficient; CI, confidence interval.

The performance of the device depends on the examiner’s technique since it is handheld. Therefore, reproducibility was verified. After receiving operating instructions, three examiners performed the measurement five times on three subjects after two or three training measurements. To evaluate the reproducibility of the device itself, positioning and marking for the measurement were performed in advance. The marking of the measurement position was excluded from the evaluation.

Table 3 shows the demographic data from 123 patients, such as age, height, weight, body mass index, and proportion of women, and the data regarding measurements of the quadriceps cross-sectional area by CT and ultrasound (in the supine and sitting positions).

Differences between quadriceps cross-sectional area obtained from images with CT and ultrasound images in the recumbent position were calculated, resulting in an absolute error for mean ± SD of 1.28 ± 3.13.

**Table 3:** Demographic and cross-sectional area data.

	Average	SD	Min.	Max.
Age (years)	78.2	8.1	46.0	92.0
Height (cm)	155.3	10.2	132.2	178.1
Weight (kg)	55.8	10.1	29.1	95.4
BMI (kg/m^2^)	23.1	3.3	15.1	33.2
US CSA (supine, cm^2^)	44.5	9.0	19.0	67.6
US CSA (sitting, cm^2^)	44.4	9.6	18.9	68.1
CT CSA (cm^2^)	43.2	10.2	20.5	72.8
Male: Female	48: 75			
Sarcopenia classification	No Sarcopenia: Sarcopenia: Severe Sarcopenia = 97: 13: 13			

SD, standard deviation; Min, minimum; Max, maximum; BMI, body mass index; US, ultrasound; CSA, cross-sectional area; CT, computed tomography.

### Correlation of quadriceps cross-sectional area between imaging methods

The unadjusted, age-adjusted, and age-sex-adjusted correlations between supine ultrasound and CT are described in Table 4. Notably, compared to CT images, coefficients were slightly more favorable when measured in the sitting position. A minimal decrease was noted in the correlation coefficients after age adjustment, and a further decrease was observed with the inclusion of age and sex adjustment.

**Table 4:** Correlation among the three types of images.

		CT	US (supine)	US (sitting)
**US (supine)**	**Not adj**	.949**	**1**	.952**
	**Age adj**	.940**	**1**	.945**
	**Auge and Sex adj**	.894**	**1**	.904**
**US (sitting)**	**Not adj**	.958**	.952**	**1**
	**Age adj**	.953**	.945**	**1**
	**Age and Sex adj**	.912**	.904**	**1**
**CT**	**Not adj**	**1**	.949**	.958**
	**Age adj**	**1**	.940**	.953**
	**Age and Sex adj**	**1**	.894**	.912**

US, ultrasound; CT, computed tomography s; adj, adjusted.

## Discussion

In this study, we introduced a novel ultrasonic muscle diagnostic device with a wide field of view that can visualize muscle tissue and evaluated its measurement reproducibility. In addition, we investigated the correlation between the quadriceps muscle cross-sectional area of CT images and ultrasound images in the same supine measurement position. Given that there was a strong correlation observed between measurements conducted in supine and sitting positions, it suggests that the quadriceps CSA can be evaluated in the same manner as that with CT even in the sitting position, in which ultrasound is easier to perform.

Regarding reproducibility, excellent results (Table 2) were obtained both within and between examiners. A good correlation was noted between the cross-sectional area measurements of ultrasound images in supine and in sitting positions, thus suggesting that there are no issues with performing the measurement in either position. The good correlation between quadriceps cross-sectional area measured in the supine position with CT and ultrasound suggests that the obtained values were accurate. However, this correlation tended to be slightly stronger between CT and US in sitting position because measurements in the sitting position allow the examiner to sit directly in front of the examinee’s thighs, thus stabilizing the examiner’s body and allowing the examiner’s wrist to smoothly move the probe from the inside to the outside of the thigh. This approach is thought to be advantageous in terms of imaging the lower part of the vastus lateralis muscle more clearly.

DXA has been considered the gold standard for muscle mass measurement when diagnosing sarcopenia [7,12] but it has the disadvantages of being expensive and requiring both specialized facilities and technicians. Therefore, it is desirable to evaluate the muscle mass using ultrasound equipment, as reported by other investigators and as demonstrated in this study, because ultrasound offers a viable alternative and is free from the aforementioned disadvantages. By evaluating muscle quality using brightness evaluation, which was not considered in this study, it will be possible to focus on muscle quality, which cannot be determined using DXA, and to measure the quadriceps muscle, which shows the most significant decrease in muscle mass due to aging.

Therefore, we believe that it is more logical to measure the quadriceps muscle, which is thought to have a large effect on the decline in activities of daily living. Previous studies have reported the usefulness of ultrasound devices in determining the thickness and brightness of the rectus femoris and vastus intermedius muscles, which are representative of the entire quadriceps [23,25]. Furthermore, a wide range of muscular assessments using ultrasound devices have been reported for the entire quadriceps [29] and the posterior aspect of the thigh [30]. On the other hand, the pattern of age-related decline in muscle mass [17,20] and its effect on knee extension strength [20] are different among the quadriceps heads. Therefore, measuring the entire quadriceps, including the intermuscular fat, by this new ultrasound technology may be necessary for detecting age-related muscle mass decline accompanying the decline in muscle strength or function, which we have recently shown using CT [22]. Evaluating the quadriceps cross-sectional area based on the standard deviation (SD) from an average obtained from young participants from the same age group or as a percentage of the young adult mean (YAM) can be a future possibility similar to how osteoporosis is currently diagnosed [31,32].

This study has some limitations. The number of cases was limited before our device was certified as a medical device; therefore, it will be necessary to include more cases in which the difference in measured values from those of CT images is large. In addition, the number of cases was too small to determine an ultrasound-based cutoff value for diagnosing sarcopenia; this is another issue to be addressed in the future. An analysis of sex-based differences is also a topic for future research. Although we showed excellent reproducibility results, we have not evaluated intra- and inter-day variations, which we also consider should be further studied. In addition, we did not report fixed or systematic errors; however, we aim to report these in our next paper.

Despite these limitations, this device has multiple advantages: measurements are simple to perform, safe, and low-cost; the device can be used anywhere; and the images can be easily understood by both the examiner and the patient at a glance. Until now, examining the muscle condition of older adults has been time-consuming and only performed by professionals, thus making it economically difficult to perform in clinical settings. However, by using this device, many patients can be examined easily within a short period. This advantage will lead to new studies being conducted to understand age-related decreases in muscle mass in older people, and this device is expected to be used as a screening tool for sarcopenia in the near future. In addition, its potential to evaluate muscle quality on the basis of image brightness opens avenues for further improvements. Comparative studies using CT images can enhance measurement accuracy, thus leading to improved diagnostic and monitoring capabilities.

## Conclusions

We introduced a novel ultrasonic muscle diagnostic device that can capture cross-sectional images of the quadriceps muscle over a wide field of view, such as in CT images, and demonstrated its usefulness in terms of measurement reproducibility and comparison with CT images in the same plane. The usefulness of the presented device was also highlighted by comparing measurements in two limb positions. In the future, we will increase the number of cases for further verification. Moreover, by adding muscle quality evaluation based on brightness and by incorporating comparisons with the values of younger people and with those of the same age group, this device is expected to be used as a screening device for diagnosing sarcopenia.

## Supporting information

S1 File (XLSX)
